# In-gel probing of individual RNA conformers within a mixed population reveals a dimerization structural switch in the HIV-1 leader

**DOI:** 10.1093/nar/gkt690

**Published:** 2013-08-08

**Authors:** Julia C. Kenyon, Liam J. Prestwood, Stuart F. J. Le Grice, Andrew M. L. Lever

**Affiliations:** ^1^Department of Medicine, University of Cambridge, Addenbrooke's Hospital, Hills Road, Cambridge, Cambridgeshire, CB2 0QQ, UK and ^2^HIV-Drug Resistance Program, Centre for Cancer Research, National Cancer Institute, P.O. Box B, Building 535, Frederick, MD 21702-1201, USA

## Abstract

Definitive secondary structural mapping of RNAs *in vitro* can be complicated by the presence of more than one structural conformer or multimerization of some of the molecules. Until now, probing a single structure of conformationally flexible RNA molecules has typically relied on introducing stabilizing mutations or adjusting buffer conditions or RNA concentration. Here, we present an in-gel SHAPE (selective 2′OH acylation analysed by primer extension) approach, where a mixed structural population of RNA molecules is separated by non-denaturing gel electrophoresis and the conformers are individually probed within the gel matrix. Validation of the technique using a well-characterized RNA stem-loop structure, the HIV-1 *trans*-activation response element, showed that authentic structure was maintained and that the method was accurate and highly reproducible. To further demonstrate the utility of in-gel SHAPE, we separated and examined monomeric and dimeric species of the HIV-1 packaging signal RNA. Extensive differences in acylation sensitivity were seen between monomer and dimer. The results support a recently proposed structural switch model of RNA genomic dimerization and packaging, and demonstrate the discriminatory power of in-gel SHAPE.

## INTRODUCTION

A variety of biochemical techniques exist to examine the secondary structure of RNA molecules via nuclease mapping (1) or chemical modification (2). Reactive sites are analysed by primer extension or 3′ end-labelling with ^32^P, separation by denaturing gel electrophoresis and autoradiographic visualization. Recently, a group of compounds have been reported that acylate the ribose 2′OH where the backbone is flexible, which preferentially occurs within single-stranded regions (3). This technique, designated SHAPE (selective 2′OH acylation analysed by primer extension), provides faster and more extensive structural mapping, as the acylating agent modifies each nucleotide irrespective of its nucleobase. Acylation sensitivity of each nucleotide is also correlated with the nuclear magnetic resonance (NMR) disorder parameter S^2^, suggesting that modification is sensitive to tertiary structure and thus may have a future role as a three-dimensional probing tool (4). Sample analysis via automated capillary electrophoresis has accelerated structural mapping, making high-throughput SHAPE vastly more powerful than previous methods (5).

These standard RNA probing techniques depend heavily on the RNA under examination existing predominantly as a single species in a unique conformation, and do not distinguish signals derived from different species within a population. It is likely that *in vivo* most RNAs adopt alternate functional conformations involving intramolecular or intermolecular interactions. Many RNAs contain structural switches, motifs that change with variations in cation concentration, or ligand binding, which in some cases mediate dimerization or multimerization (6–9). These RNAs often exist in solution in a mixture of conformers, thus making it necessary to manipulate buffer composition, sample concentration or nucleotide sequence to stabilize and probe individual conformers. The use of non-physiological conditions is not guaranteed to generate native *in vivo* structure, and where it is necessary to mutate the wild-type sequence to disrupt interactions or structures, off-target effects can be an impediment.

The ability to interrogate individual RNA structures within an initially mixed population would therefore improve the accuracy, sensitivity and speed of secondary structure modelling and help reveal dynamic and functional changes. Here, we present such an approach, designated in-gel SHAPE, where a mixed structural population of RNAs is separated by native gel electrophoresis and probed within the gel matrix, to determine the structures of individual conformers without the need for mutagenesis or variation of refolding conditions. In-gel probing to examine the folding pathway of the *Tetrahymena* self-splicing intron using dimethyl sulphide (DMS) and kethoxal has been reported (10). In-gel SHAPE can be used to visualize similar processes, but has particular advantages: it is performed on unlabelled RNA and allows simultaneous probing of all four nucleobases; in addition, separation of complementary DNAs by capillary electrophoresis enables acquisition of data from much longer reads. This combination makes in-gel SHAPE a simple and facile technique to use to study RNA structures.

We have validated this technique using a well-characterized stem-loop structure and thereafter used it to examine HIV-1 RNA monomers and dimers. The ∼9-kb HIV-1 RNA genome dimerizes via a palindromic site within the 5′ untranslated region (UTR) designated the dimerization initiation site (DIS, 8). The process is crucial to viral replication and is a prerequisite for successful packaging of the diploid genome into the assembling particle to form an infectious virion (11). In HIV, a dimeric RNA is also necessary for correct proteolytic processing of the Gag polyprotein during virion maturation (12,13). Gag is responsible for selecting and encapsidating the correct RNA into the developing virion but it is not known whether Gag specifically packages a dimer or two monomers that subsequently dimerize (8). Visualizing the structures of the monomer and dimer separately, as well as their interactions with Gag, is a critical element in understanding the HIV-1 lifecycle. The 5′ region of the RNA containing the DIS, together with recognition sequences and structures to which Gag binds, constitutes the packaging signal (14). Previous structural studies of this region, using biochemical and/or phylogenetic approaches, or examining individual stem-loops by three-dimensional methods, have generated a range of alternative models (15–19). Some conclude that the structure of one-half of the dimer is identical to the monomer with the exception of base pairing at the DIS (5,20). Alternative models propose that monomeric RNA adopts a different secondary structure from the dimeric RNA across several parts of the molecule (21,22).

Using in-gel probing of the HIV-1 packaging signal monomer and dimer, we show significant differences across much of the structure, but consistent low acylation sensitivity at the DIS. This implies a structural switch mechanism where the palindromic DIS is base-paired intramolecularly to another sequence in monomeric RNA and where it interacts with its counterpart intermolecularly in the dimer. When our data are compared with previous studies, they most closely fit a model where, in the monomer, the DIS base pairs intramolecularly form a pseudo-knot with part of the U5 region (22).

## MATERIALS AND METHODS

### RNA preparation

RNA was prepared by *in vitro* transcription from DNA templates containing a T7 RNA polymerase promoter at their 5′ ends. Templates were prepared by polymerase chain reaction using 1× Biomix red (Bioline), 0.5 µg of plasmid DNA (pSVC21, 23) and 2 µM primers (forward, TAATACGACTCACTATAGGGTCTCTCTGGTTAGACCAGATCTG; reverse, CTTTCCCCCTGGCCTTAACC: TAR forward, TAATACGACTCACTATAGGCCTTCGGGCCAAGGTCTCTCTGGTTAGACC; TAR reverse, CACTACTTGAAGCACTCAAGG). Products were visualized and purified by electrophoresis on 1% agarose gels in Tris-borate ethylenediaminetetraacetic acid (TBE) (89 mM Tris base, 89 mM boric acid and 2 mM ethylenediaminetetraacetic acid (EDTA)), staining with 1.3 µM ethidium bromide and subsequent gel extraction (Qiagen gel extraction kit, following manufacturer’s instructions). *In vitro* transcriptions were performed using the MEGAscript T7 kit (Applied Biosystems) according to the manufacturer’s instructions. Each 20-μL reaction contained 7.5 mM deoxyribonucleotide triphosphates (dNTPs), 1× reaction buffer, 2 μg of template DNA and 2 μL of T7 RNA polymerase for 3 h at 37°C. DNA templates were degraded for 60 min at 37°C with 4 U of DNase (TURBODNase, Applied Biosystems). RNAs were purified on MEGAclear columns (Applied Biosystems) according to the manufacturer’s instructions, eluted in water and stored at −20°C.

### In-gel chemical acylation of RNAs

Thirty micrograms of RNA was resuspended in 200 µl of renaturation buffer (10 mM Tris pH 8, 100 mM potassium chloride (KCL) and 0.1 mM EDTA), heated at 85°C for 5 min and slow-cooled to room temperature. Fifty microliters of refolding buffer was added to adjust the buffer to 40 mM Tris pH 8, 4 mM magnesium chloride (MgCl_2_), 130 mM KCl and incubated at 37°C for 30 min. One-fifth volume of the native loading dye (40% (v/v) glycerol, 44 mM Tris-borate pH 7 and 0.25% orange G dye) was added, and 12 equal samples were loaded onto lanes of a 20 cm × 18.5-cm 4% acrylamide non-denaturing gel prepared with 1× tris-borate magnesium (TBM) (89 mM Tris base, 89 mM boric acid and 0.1 mM MgCl_2_) and fractionated at 100 V for 6 h at 4°C. A gel fragment corresponding to an RNA ladder (RNA Century Plus, Ambion) and one RNA sample was excised and stained for 10 min with 1.3 µM ethidium bromide in 1× TBM and visualized under ultraviolet. The ethidium bromide-stained gel slice and unstained gel were aligned, and specific unstained bands were removed using a scalpel. Each gel piece was divided into two equal parts, with one fragment soaked in 1× TBM containing 10% dimethyl sulphoxide, and one containing 10% 100 mM *N*-methylisatoic anhydride (NMIA, in dimethyl sulphoxide) and placed at 37°C for 45 min. Gel slices were washed three times in 1× tris-acetate EDTA (TAE) (40 mM Tris-acetate and 1 mM EDTA pH 8.3), crushed and placed into an Elutrap (Whatman) channel containing 1× TAE. RNA was electroeluted overnight at 100 V into a chamber bordered by Whatman BT-1 and BT-2 membranes, then precipitated overnight at −20°C with 300 mM sodium acetate and 2.5 volume of ethanol. RNA was recovered by centrifugation, washed with 70% ethanol and resuspended in 10 µl of water. RNA concentration was determined by spectrophotometry.

### Reverse transcription and analysis

Four picomoles of RNA was resuspended in 12 µl of 2.1 mM Tris pH 8.0, 42 µM EDTA, containing 5 nmol of 6FAM^TM^-labelled primer (Applied Biosystems) for NMIA-modified samples and VIC®-labelled primer (Applied Biosystems) for negative control samples. Primers were annealed to the RNA at 85°C for 1 min, 60°C for 5 min, 35°C for 5 min and extended for 50 min at 55°C following addition of 8 µl of extension mix [100 U of Superscript III reverse transcriptase (RT), Invitrogen; 1.5× SSIII RT buffer, Invitrogen; 12.5 mM dithiothreitol (DTT), 1.25 mM Deoxycytidine triphosphate (dCTP), deoxyadenosine triphosphate (dATP), deoxyuridine triphosphate (dUTP) and 7-deaza-deoxyguanosine triphosphate (dGTP)]. Complementary DNAs were combined and RNA was degraded with 200 mM sodium hydroxide (NaOH) at 95°C for 3 min, followed by cooling on ice and the addition of two sequencing ladders. Sequencing ladders were prepared using the same primers, labelled with NED^TM^ and PET® (Applied Biosystems), and cycle sequencing was performed using a DNA sequencing kit (Sequitherm Excel II, Epicentre or DNA cycle sequencing kit, Jena). Each reaction contained 50 ng of DNA template, 1.3 nmol NED^TM^- or PET®-labelled primer, and 1× sequencing buffer, extension mix and enzyme as per manufacturer’s instructions. Cycle sequencing was performed at 95°C for 5 min followed by 30 cycles of 95°C for 30 s, 37°C for 30 s and 70°C for 1 min in a Multigene thermocycler (Labnet). Samples were resuspended in 30 µl of formamide and fractionated by capillary electrophoresis (Applied Biosystems 3730xl analyser). SHAPEfinder software was used to align the sequences and calculate the relative areas of each peak as in (24). The areas of negative control peaks were subtracted from the NMIA-modified RNA peaks, and data were normalized by dividing the value at each nucleotide position by the average of the top 8% of results below data points above the third quartile plus 1.5× the interquartile range. Mobility shift controls for each primer were carried out as described (24).

### Structural modelling

Modelling was carried out using RNAstructure (25). Where SHAPE data were used as part of the modelling they were entered as soft pseudo-free energy constraints. Structures were illustrated in XRNA.

## RESULTS

### In-gel probing of RNA generates accurate reproducible results

**Figure 1.**
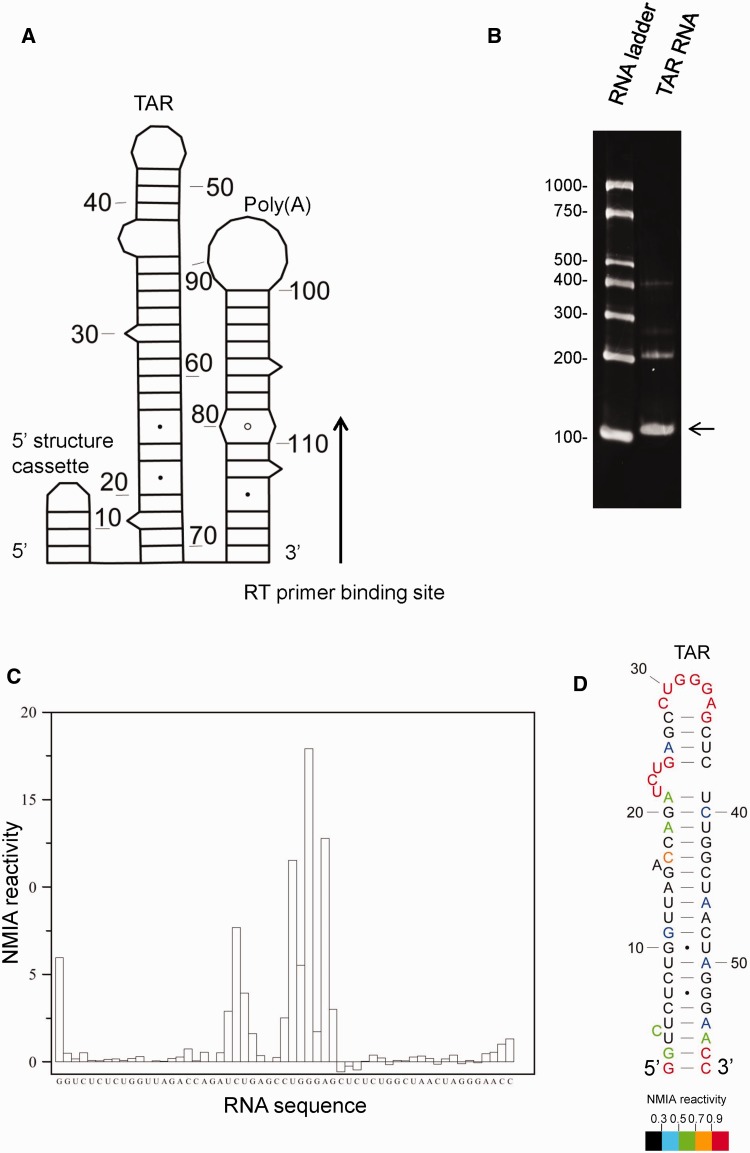
In-gel SHAPE probing of a well-characterized stable RNA structure generates accurate reproducible results. (**A**) HIV-1 TAR RNA was appended with a 5′ structure cassette and poly(A) stem-loop. Nucleotides are numbered every 10 bases. (**B**) Ethidium bromide-stained native polyacrylamide gel. Arrow shows the monomeric TAR band that was excised for in-gel structural analysis. (**C**) NMIA reactivity trace of the in-gel probed monomeric TAR RNA. (**D**) RNAstructure prediction of TAR RNA, using in-gel SHAPE data as pseudo-free energy constraints. Individual NMIA reactivities are shown using different colours. Nucleotides are numbered every 10 bases.

The HIV-1 TAR (*trans*-activation response) element, previously characterized by NMR, X-ray crystallography (26) and SHAPE (4), was selected as a suitable RNA for validating in-gel probing. To facilitate SHAPE analysis of the entire stem-loop, we added the poly(A) stem-loop as a 3′ structure cassette, and a previously published 5′ structure cassette (3). Neither addition was predicted to disrupt the TAR structure as judged by minimal free-energy modelling using RNAstructure: the construct and structural prediction is shown schematically in Figure 1A. *In vitro* transcribed RNA was purified, renatured and fractionated in each well of a 4% polyacrylamide gel under native conditions, alongside an RNA ladder. The ladder and one lane of the RNA sample were excised with a scalpel, soaked in running buffer containing ethidium bromide for 5 min and visualized by ultraviolet transillumination (Figure 1B). The unstained gel was aligned, and a band corresponding to the position of migration of the monomeric RNA, but slightly wider, was excised from the remainder of the lanes on the gel (illustrated in Supplementary Figure S1). RNA was probed *in situ* with NMIA, recovered from the gel, reverse transcribed and analysed as described in Materials and Methods. Seven independent experiments were performed, and on average, the SHAPE reactivity category (<0.3, 0.3–0.5, 0.5–0.7, 0.7–0.9, >0.9) was the same at each nucleotide in more than five of these, showing the results are highly reproducible. NMIA reactivity profiles of the monomeric TAR hairpin are shown in Figure 1C and by colour in Figure 1D. Numerical values are given in Supplementary Figure S2. Reactivities were used as pseudo-free energy constraints in the RNAstructure prediction algorithm to examine the TAR hairpin (shown in Figure 1D). The reactivities and resulting structural prediction correspond closely with previously published structures. The 57 nucleotide (nt) stem-loop contains unreactive helices and an apical loop region and 5′ UCU bulge that are highly NMIA-reactive, as evident in Figure 1C. A similar reactivity pattern was previously observed by SHAPE, using 1-methyl-7-nitroisatoic anhydride as the acylating agent (4). Examining the structure in detail, each apical loop nucleotide is highly reactive, as is the 3′ G of the closing pair, reflecting the previously reported conformational flexibility and disorder of nucleotides in the loop (27). The UCU bulge is also highly NMIA reactive. Although these nucleotides are not canonically paired, the 3′ U and C have previously been shown by NMR and X-ray crystallography to be stacked, an interaction which often reduces NMIA reactivity (28). However, X-ray crystallography of this region shows that nucleotides in this internal loop have sugars that do not adopt the classical C3′-endo conformation characteristic of A-form RNA, i.e. U25 is C2′-endo whereas U23 and G26 are C2′-exo-C3′-endo (26). These non–A-form backbone conformations can be highly reactive to chemical acylation (29). Minimal reactivity is detected in nucleotides within the helices, confirming their previously observed A-form structures (26). Some reactivity exists at the extreme 5′ and 3′ closing pairs, which is commonly seen in SHAPE (30). Taken together, these data indicate that in-gel SHAPE is accurate and reproducible. It should also be noted that the RNA concentrations used for gel electrophoresis and ethidium bromide visualization induce higher order structures that may be dimers (bands at ∼200 nt, Figure 1B). In-gel SHAPE, however, avoids signals associated with multimeric RNAs, enabling unambiguous examination of the monomer.

The conditions we used for in-gel SHAPE were chosen to closely resemble those previously optimized for *in vitro* probing, taking into account the hydrolysis time of NMIA in aqueous solution (5,9). Therefore, temperature (37°C) and time of incubation were not varied once we ascertained that these standard reaction conditions also worked accurately within the gel matrix. However, it was necessary to optimize other parameters: the RNA concentration was determined by how much RNA can be readily visualized by ethidium bromide staining, and we noticed consistently lower recovery yields of RNA that had been modified with NMIA prior to electrophoretic separation (data not shown). In an effort to increase recovery of NMIA-modified RNA from the matrix, we performed passive overnight elution in 300 mM sodium acetate at 37°C, with shaking, but yields were even lower (data not shown). We also prepared gels using *N-N’* diallyltartardiamide (31), in place of bisacrylamide, and dissolved the gel in 2% periodic acid instead of recovering the RNA by electroelution. However, we found the gel more difficult to manipulate and could not recover high levels of pure RNA from the dissolved gel (data not shown). Attempts to modify the RNA with a lower concentration of NMIA showed little reactivity above the negative control (5 mM NMIA, Supplementary Figure S3A, compared with the standard 10 mM NMIA probing shown in Supplementary Figure S3B). When examining 100–500-nt RNAs, the yield remained consistent at ∼40%, giving sufficient recovery for multiple primer extension reactions (which require ∼4 pmol of RNA) from each gel. However, recovery yields dropped when eluting larger molecules, to ∼3% of an 851-nt RNA (Supplementary Figure S4A). Increasing electroelution time did not recover significantly more RNA, with 16 h being the optimal period (Supplementary Figure S4B). Probing with 1M7 for 3 min in place of NMIA also yielded accurate results when TAR structure was examined as before (Supplementary Figure S4C). Data shown were from RNA probed with 10 mM 1M7 and are an average of four replicates; probing with 5 mM 1M7 also yielded similar results, but data were more variable between replicates (not shown); hence we recommend probing with 10 mM 1M7.

### In-gel SHAPE supports a structural switch model of HIV-1 RNA dimerization

**Figure 2.**
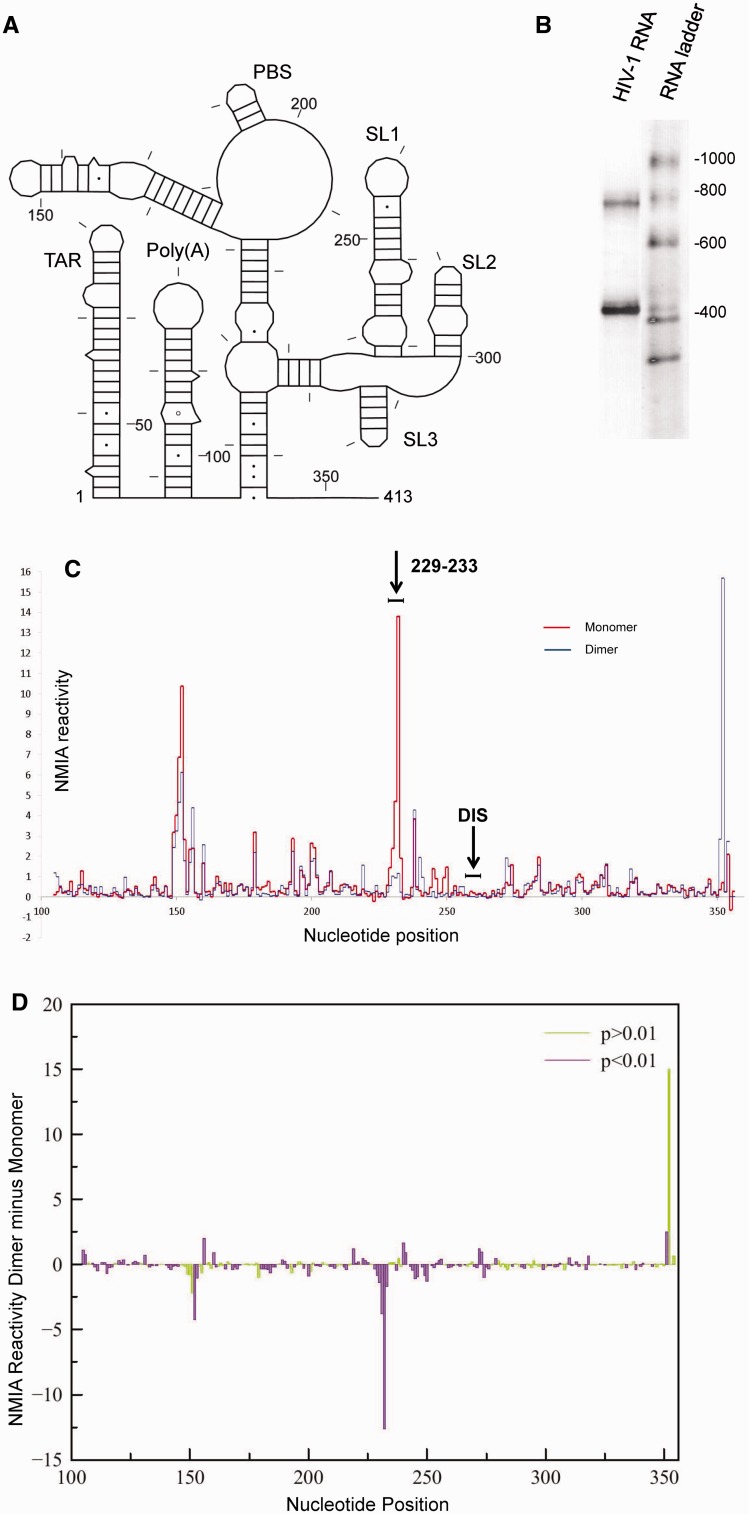
In-gel SHAPE of monomeric and dimeric HIV-1 packaging signal RNA shows significant differences in reactivity between monomer and dimer. (**A**) Schematic diagram of the HIV-1 RNA examined. Nucleotides are numbered every 50 bases and marked every 10 bases. (**B**) Ethidium bromide-stained polyacrylamide gel slice showing HIV-1 RNA monomer and dimer excised for probing. (**C**) NMIA reactivity of monomer (red) and dimer (blue) for nucleotides 100–354. The position of the 6 nt DIS is marked above. Results are an average of 7–10 independent experiments. (**D**) Plot of average SHAPE reactivity of the monomer subtracted from the average NMIA reactivity of the dimer at each nt position. Colour shows statistical significance by *t*-test, where purple bars are statistically significant and green are not significant, to *P* < 0.01.

As in-gel SHAPE can probe individual RNAs of different electrophoretic mobility within a mixed population, it follows that it could be used to examine conformational switches or dimerization processes. The HIV-1 RNA genome dimerizes via palindromic sequences (GCGCGC, GUGCAC or GUGCGC (32)) within the 5′ UTR, designated the DIS. Several structural models have been proposed for the monomeric and dimeric RNAs, often using mutagenesis as a means of isolating and probing the structure of a monomer, rather than by isolating a purely monomeric population of wild-type sequence (20,33). Many studies have concluded that the DIS is exposed in the monomer, and that the overall structure of a monomer is similar to one-half of the dimer (5,17,20). Alternative structural switch models suggest that in the monomer the DIS is paired with a 5′ sequence (21,22). Because in-gel SHAPE distinguishes individual RNA structures without the need for mutagenesis, the wild-type monomeric and dimeric populations were studied. The packaging signal (nts 1–413 of the HIV-1 genome, shown schematically in Figure 2A), prepared by *in vitro* transcription, was renatured and fractionated by native gel electrophoresis. The stained portion of the gel is shown in Figure 2B. Bands corresponding to the monomer (400 nt) and the dimer (800 nt) are visible. Unstained monomer and dimer were excised and probed with NMIA as described above. These initial renaturation conditions used previously published buffers (34) and were chosen to produce relatively equal amounts of monomer and dimer. SHAPE reactivity data are given in Supplementary Figure S5 and illustrated for nts 100–354 in Figure 2C. Data are an average of 7–13 replicates at each nucleotide position. The experiment was extremely reproducible; each nucleotide showed equivalent NMIA reactivity in >70% of replicates for the monomer and 79% of replicates for the dimer. This corresponded to an average standard deviation of 0.29 for the monomer and 0.22 for the dimer for nucleotides with reactivity <0.9. The standard deviation was higher among the most reactive category of nucleotides.

Comparing the SHAPE profiles of the monomer and dimer reveals that the DIS, corresponding to nts 257–262, is unreactive in both (Figure 2C). There is a large difference at nts 229–233, which exhibit extremely high reactivity in the monomer but are intermediate to high reactivity in the dimer, despite their location within a helix. Dimerization can be regarded as a two-step process, with ‘loose’ dimers converting to ‘tight’ dimers (8). Loose dimers readily revert to a monomeric form, and as these nucleotides are so highly acylation sensitive in the monomer (with a range of 1.4–13.8), destabilization of a small fraction of the dimers would account for the acylation sensitivity seen at these positions (0.23–1.15). The region forming SL2 and SL3 has a similar reactivity profile in both species (nts 282–325).

Many of the differences in reactivity between the monomer and dimer are statistically significant (Figure 2D). The largest significant difference is within nts 229–233. The reactivity peak at the 3′ end of the structure is not statistically significant, as data for nucleotides closest to the reverse transcription primer are more variable. However, it is not just the large NMIA reactivity differences that are statistically significant. The in-gel SHAPE results are sufficiently reproducible that even small differences in reactivity between monomer and dimer can be detected. The results imply that although some of the secondary structural elements are broadly similar between monomer and dimer, their backbone conformations both within stem and loop regions are slightly different. Low reactivity of the DIS in both the monomer and dimer, together with the other large differences in reactivity across the structures, makes it unlikely that the structural differences between a monomer and dimer consist of simply the pairing or unpairing of DIS nucleotides. Rather, our data support a structural switch mechanism involving changes in several regions of the RNA.

When NMIA reactivity profiles of the monomer and dimer are entered as pseudo-free energy constraints in RNAstructure, a range of models with similarity to the long distance interaction-branch multiple hairpin (LDI-BMH) switch proposed by Abbink and Berkhout (21) are generated (data not shown). However, the RNAstructure algorithm, using SHAPE data as constraints, is unable to predict pseudo-knots, such as the one recently proposed to form in the monomer (22). Although the vast majority of nucleotides would be predicted to have a similar SHAPE profile in each of these structural switch models, even in regions that change structure between a monomer and a dimer, there are a small number of nucleotides that would be paired or unpaired in one model and not in the others. Our SHAPE data differences between the monomer and dimer are most consistent with the structural switch involving the DIS/U5 pseudo-knot (22). The large reactivity difference at nts 229–233 is less in keeping with the LDI-BMH switch model, as this region is static in structure between monomer and dimer, making this magnitude of reactivity change unlikely. The reactive U65 (1.48 in the monomer and 1.26 in the dimer, Supplementary Figure S5) is also proposed to be within the stable R-Gag duplex in the LDI model, whereas it is adjacent to a bulge in the Lu *et al.* model (22). For comparison, NMIA reactivity data mapped onto the LDI model are shown in Supplementary Figure S6.

### The dimer structure is similar to previously proposed models

**Figure 3.**
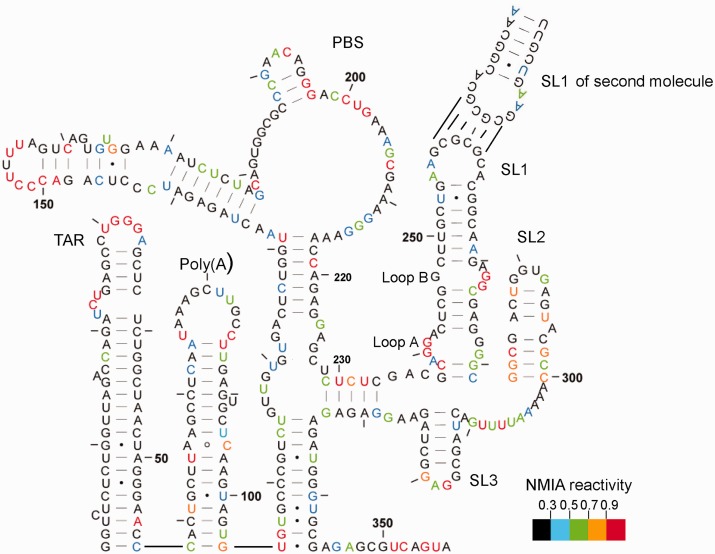
In-gel SHAPE data mapped onto the dimeric RNA structure proposed by Lu *et al.* (22). RNA was renatured as in Materials and Methods and probed with 10 mM NMIA. Nucleotides are numbered every 50 bases and marked every 10 bases. Reactivities represented by each colour are shown in the key. Data are an average of seven independent experiments.

**Figure 4.**
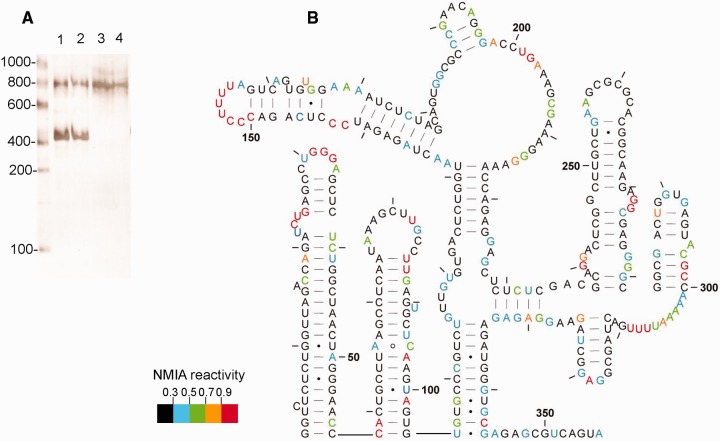
In-gel SHAPE data from HIV-1 RNA renatured using snap-cooling refolding conditions. (**A**) Ethidium bromide-stained gel slice showing the relative amounts of monomer and dimer present when RNA is renatured by slow-cooling as described in Materials and Methods (lanes 1 and 2), or using Lu *et al.* conditions (22) (lanes 3 and 4). (**B**) NMIA reactivities mapped onto the proposed structure, shown by colour according to the key. Data are an average of four independent experiments.

NMIA reactivities at individual nucleotides are mapped onto a model proposed for the dimer by Lu *et al.* (22) in Figure 3 and, with minor variations, are largely compatible. Examining the dimer reveals a TAR stem-loop with low reactivity within the stem and a highly reactive terminal loop and UCU bulge, along with a reactive A residue near the base of the stem opposite the single nucleotide bulge. Reactivity to SHAPE reagents is not uncommon in the closing pairs of helices or in the adjacent nucleotides (30). NMIA reactivities likewise map accurately onto the poly(A) stem-loop and the U5-AUG interacting helix, as well as within the PBS region. Low reactivity of the semi-palindromic sequence 181-GUGGCGC-187 in the dimer suggests that this may act as a second dimerization contact, particularly as this sequence is more reactive in the monomer. However, when the RNA is rendered monomeric using a locked nucleic acid (LNA) complementary to the DIS and predicted to stabilize a ‘hemidimer’ reactivity of this region does not significantly increase (35), suggesting an intramolecular rearrangement. This region of the UTR has the greatest heterogeneity in predicted structure in currently published models (36). Nucleotides 229–233 pair with 329–333 to form a 5-bp helix. Although the 5′ side of this helix displays intermediate to high reactivity, this is 10-fold lower than observed for the equivalent region of the monomer (highlighted by arrow to 229–233 in Figure 2C), possibly reflecting destabilization of the dimer during probing. Any ‘loose’ dimer formed may convert to a monomer and, as this region is so highly reactive in the monomer, even a small loss of dimerization could have a large impact on the average NMIA reactivity in this region. Our 3D analysis of the region also suggests it would be possible for interactions to occur with the neighbouring helix at nts 125–131 and 217–223 (35).To ascertain whether reactivity of nts 229–233 reflects interconversion of dimer to monomer, we renatured the RNA using the method of Lu *et al.*, which gave us 90–100% dimer (Figure 4A), and used in-gel probing to determine NMIA reactivities. Reactivities at this helix are significantly lower, and the data fit onto the proposed dimeric structure accurately.

**Figure 5.**
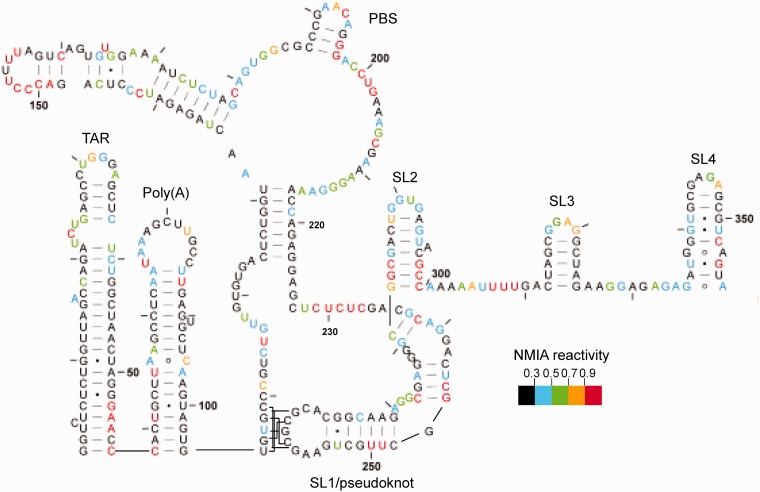
In-gel SHAPE data from monomeric HIV-1 RNA mapped onto the proposed pseudo-knot structure. RNA was renatured using slow-cooling conditions, as in Materials and Methods, and probed using 10 mM NMIA. Reactivities are shown by colour according to the key.

The C219 is within a helix but NMIA-reactive in the less stable dimer, as shown in Figure 3. It is, however, unreactive in the monomer (Figure 5) and in the more stable dimer (Figure 4B). Both structural switch models propose that this nucleotide is within an identical 7-bp helix. Occasionally, nucleotides within helices are reactive if maintained in a non–A-form conformation (28), and this region may be involved in the refolding pathway between monomer and dimer. Stem-loops 1, 2 and 3, comprising the major packaging signal, fit the SHAPE data accurately.

### The derived monomeric structure fits most closely with a recently proposed pseudo-knot–containing model

SHAPE reactivity data for the monomer are shown in Figure 5. Again, the data map accurately onto the TAR and poly(A) structures, although the loop regions are less reactive and there is more reactivity in the stems around the bulges and GU pairs, suggesting that the base of the TAR hairpin is less stable in the monomer than in the dimer. Even lower reactivity in the dimer when renatured to give almost 100% dimer (Figure 4) supports this observation. Other groups have found a necessity for a stable TAR structure in dimerization, but the loop region was recently shown not to participate (37); our data suggest that the base of TAR rather than the bulge and loop regions may be involved in regulating the monomer–dimer structural switch. Nucleotides 105–116, which form the 5′ side of the U5-AUG helix in the dimer, are less reactive at the 5′ end and more reactive at the 3′ end, supporting the pseudo-knot model in which the 5′ end is paired to the DIS, with fewer GU pairs than in the U5-AUG helix, and the 3′ end is single-stranded. The single-stranded regions at 121–122 and 226–227 may be paired, as our data show low reactivity in this region; however, sequence variation would explain the single-stranded nature of the region in other isolates such as that examined by Lu *et al.* (22). NMIA reactivities for the PBS stem-loops map once again accurately onto the structure, and as previously mentioned the CUCUC region at nt 229–233 is reactive, and is now single-stranded. Reactivities within the stem of SL1 have changed considerably, indicating that the pseudo-knot is likely to disrupt this entire structure, so the monomeric structure is likely to differ from the Lu *et al.* model in this respect. SL2 is also more reactive at the base than it is in the dimer, and reactivities again map accurately onto SL3 and onto SL4. As there are few nucleotides that are paired in the pseudo-knot model but not in the LDI model (21), or vice versa, the LDI model is shown for comparison in Supplementary Figure S6. Reactivity at U65 makes the formation of a helix at this point, which is crucial to the LDI model, unlikely.

## DISCUSSION

In-gel SHAPE is a rapid, accurate and facile method of probing and modelling individual RNA conformers, or of deciphering mixed probing signals. Our validation, using the TAR RNA, shows that it is highly reproducible, and recapitulates data from a variety of secondary and tertiary structural methods. Examining RNAs migrating individually within a native gel is expected to eliminate artefacts in the data from aberrantly folded molecules and low levels of transient interconverting conformers. SHAPE analysis of the HIV monomer/dimer mixture before electrophoresis produced nucleotide reactivity inconsistent with one species or the other (data not shown). Where RNAs of interest migrate differently enough to be separated on a gel, it allows probing of two or more individual structures within the same mixture. Its uses could extend to the examination of structural switches, RNA multimerization or RNA structure within RNA–protein complexes.

We hypothesized that in-gel SHAPE could facilitate resolving the native structures of the HIV-1 packaging signal monomer and dimer without the need for mutagenesis to stabilize either conformation. The structure of the HIV-1 packaging signal RNA dimer, investigated by in-gel SHAPE, is almost identical to previously published structures of the monomeric RNA, with base pairing between the two molecules across the DIS (5, and BMH structure in 21). Reactivity profiles correspond closely with the proposed model, and the higher reactivities of some nucleotides in the dimer refolded under conditions that promote both monomer and dimer formation, compared with conditions that strongly favour formation of the dimer, illustrate nucleotides that may adopt different structures in the folding pathway. C219 is reactive, yet within a helix. Some non–A-form backbone angles have been shown to increase reactivity to SHAPE reagents (28), which may be the case with C219. The pairing sequence, nts 125–131, is also known as the primer activation signal (PAS). The first step in HIV genome replication involves annealing of tRNAlys3 to the primer binding site to serve as the primer for (−) strand DNA synthesis. This event is facilitated by an interaction between the PAS and the anti-PAS motif in the tRNA (38). Mutations in the C219 region of the HIV-1 genome that disrupt the ability of these two helices to pair, thus exposing the PAS, enhance the rate of reverse transcription (39). Therefore, conformational flexibility of this helix may be vital for loading of the tRNA primer onto the HIV-1 template. There are also many small but statistically significant differences in backbone NMIA reactivities between the monomer and dimer across the PBS, suggesting that although the overall secondary structures are similar, the tertiary configuration of the RNA across the PBS likely differs between monomer and dimer. Different probing reactivities have been observed in this region by other groups (for example 5,17,40), which may reflect examination of different viral isolates, mixed structural populations or the presence of tRNA. It is unknown whether the tRNA is loaded onto monomeric or dimeric RNA, but the structural differences in this region may guide tRNA loading at the correct point in the lifecycle. It has been recently shown that the U-rich loop at nts 151–157 mimics the human tRNA Lys anti-codon domain, allowing the tRNA to anneal to the HIV-1 genome (41). Nucleotides of this loop show equivalent reactivities in both monomer and dimer, with U154 and A157 unreactive and the other nucleotides exhibiting strong reactivity. Significantly higher reactivities in these nucleotides of the monomer may have implications for the tertiary structure and recognition by the tRNA. Jones and colleagues noted that SL1 must be present for efficient interaction between the lysyl tRNA-synthetase and the HIV-1 RNA, implying a role for the monomer–dimer structural switch in tRNA loading (41).

SLs1–3 are designated the major packaging signal region and contain the majority of high-affinity Gag binding sites. SL3 in particular has been shown to bind Gag specifically, and this interaction is thought to be an initial step of the packaging process (14). However, equivalent SHAPE profiles for SL3 nucleotides of monomeric and dimeric RNA suggest the structure of this stem-loop is static; hence it is unlikely that Gag would be able to differentiate between monomeric and dimeric RNA by binding to SL3 alone. Instead, the orientation of SL3 in the context of the whole region may be important for Gag recognition of either monomeric or dimeric RNA. The nucleocapsid domain of Gag has been shown to contact the loop of SL3, but there may be interactions between other domains and different regions of the RNA (42,43). Formation of the 5-bp helix subtending the major packaging signal region at nts 229–233 and 329–333 may be a crucial part of this structural alignment. In addition, SL1, which contains the DIS at its tip, displays different SHAPE profiles across the entire stem and internal loop regions between monomer and dimer. In dimeric RNA, it appears to be a stable stem-loop with the two internal loops, A and B (shown in Figure 3), showing strong NMIA reactivity, fitting the NMR-solved structure of SL1 in isolation (44). In the monomer, there is more reactivity within the stem and lower reactivity within these loops, suggesting that remodelling of SL1 is more extensive than that previously shown (22). This again may have implications for packaging, as SL1 has been shown to contain nucleocapsid binding sites (5), and loop A is an interaction site for the Rev protein (45). Although the primary function of Rev is to mediate nuclear export of unspliced or partially spliced RNAs, a Rev–loop A interaction has also been shown to facilitate efficient genome packaging (46). Our results imply that loop A is only formed stably in the context of the dimer; hence this might be a mechanism by which the virus selectively packages dimeric RNA. Lower acylation sensitivity of SL1 loop B and the SL4 tetraloop in the monomer are consistent with a previously proposed interloop interaction (47).

## SUPPLEMENTARY DATA

Supplementary Data are available at NAR online.

## FUNDING

Biomedical Research Centre and the Medical Research Council [G0800142 to A.L.] and the Intramural Research Program of the National Institutes of Health, National Cancer Institute [to S.Le G.]. Funding for open access charge: Medical Research Council [G0800142].

*Conflict of interest statement.* None declared.

## Supplementary Material

Supplementary Data
