# Supplementary material for: In-gel probing of individual RNA conformers within a mixed population reveals a dimerization structural switch in the HIV-1 leader
Source: Nucleic Acids Res. 2013 Aug 8;41(18):e174. doi: 10.1093/nar/gkt690 (PMC3794615; doi:10.1093/nar/gkt690)
Supplement: Supplementary Data [file supp_41_18_e174__index.html]

In-gel probing of individual RNA conformers within a mixed population reveals a dimerization structural switch in the HIV-1 leader — In-gel probing of individual RNA conformers within a mixed population reveals a dimerization structural switch in the HIV-1 leader — Supplementary Data 

# In-gel probing of individual RNA conformers within a mixed population reveals a dimerization structural switch in the HIV-1 leader

## Supplementary Data

files

**Files in this Data Supplement:**

- Supplementary Data - pdf file
