# Supplementary material for: In-gel probing of individual RNA conformers within a mixed population reveals a dimerization structural switch in the HIV-1 leader
Source: Nucleic Acids Res. 2013 Aug 8;41(18):e174. doi: 10.1093/nar/gkt690 (PMC3794615; doi:10.1093/nar/gkt690)
Supplement: Supplementary Data [file supp_gkt690_nar-00836-met-g-2013-File010.pdf]

Supplementary Figure 1

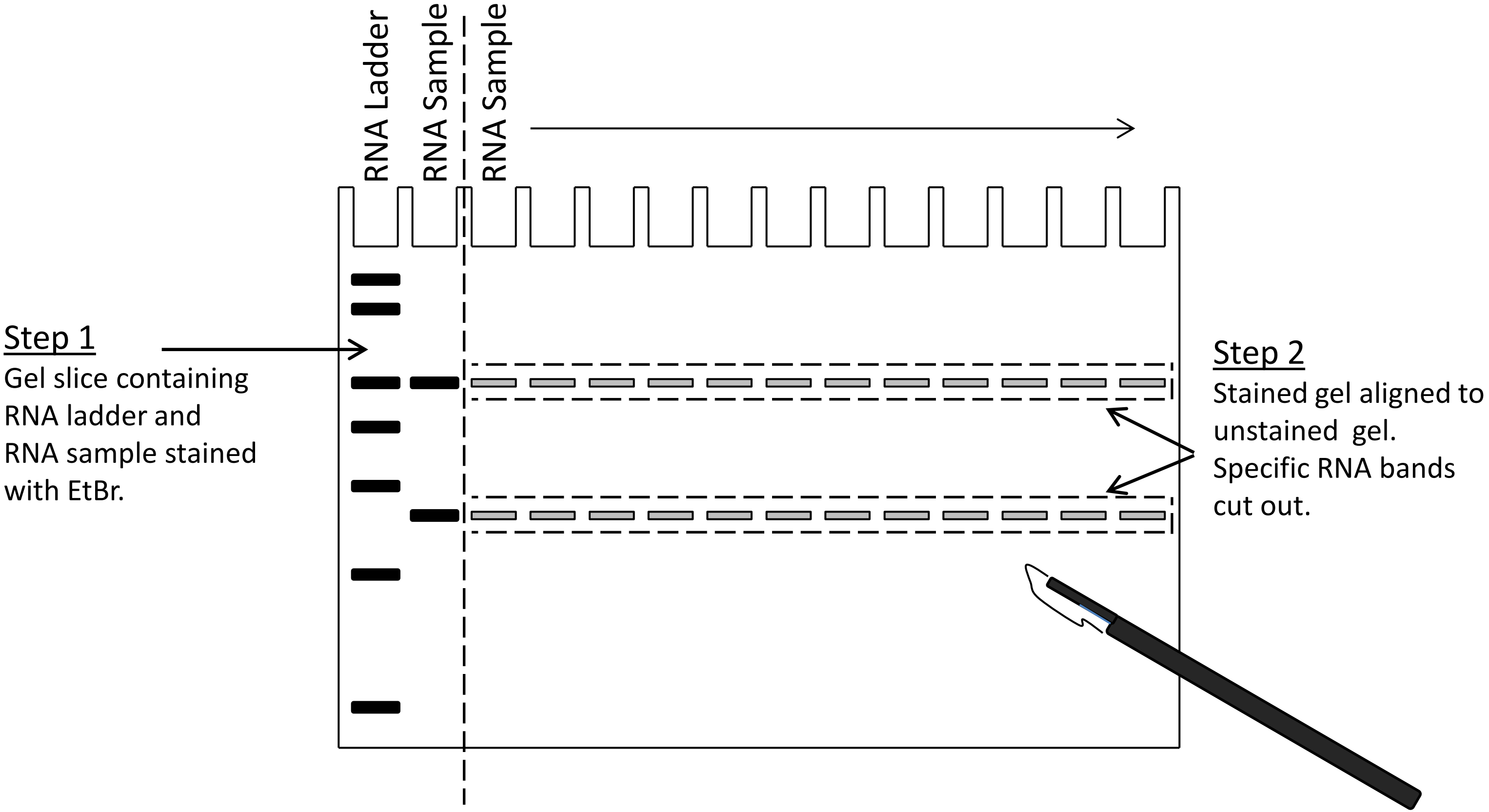

Supplementary Figure 2: in-gel SHAPE reactivity data for TAR

G	5.953604
G	0.503008
T	0.155143
C	0.525321
T	0.094119
C	0.057418
T	0.14074
C	0.163049
T	0.076017
G	0.199565
G	0.314613
T	-0.00073
T	0.066686
A	0.037576
G	0.195488
A	0.266484
C	0.739813
C	0.054989
A	0.549049
G	0.010771
A	0.507581
T	2.884858
C	7.680767
T	3.938523
G	1.614759
A	0.366296
G	0.002124
C	0.235484
C	2.508034
T	11.53434
G	5.502876
G	17.90891
G	1.718674
A	12.78207
G	2.99335
C	-0.56295
T	-0.25171
C	-0.46321
T	0.041931
C	0.388936
T	0.233906

G	-0.15058
G	0.088095
C	0.06324
T	0.273319
A	0.332222
A	0.191994
C	-0.14327
T	0.172373
A	0.391972
G	-0.11951
G	0.076485
G	-0.03847
A	0.480063
A	0.558578
C	1.002313
C	1.300829

Supplementary Figure 3

A

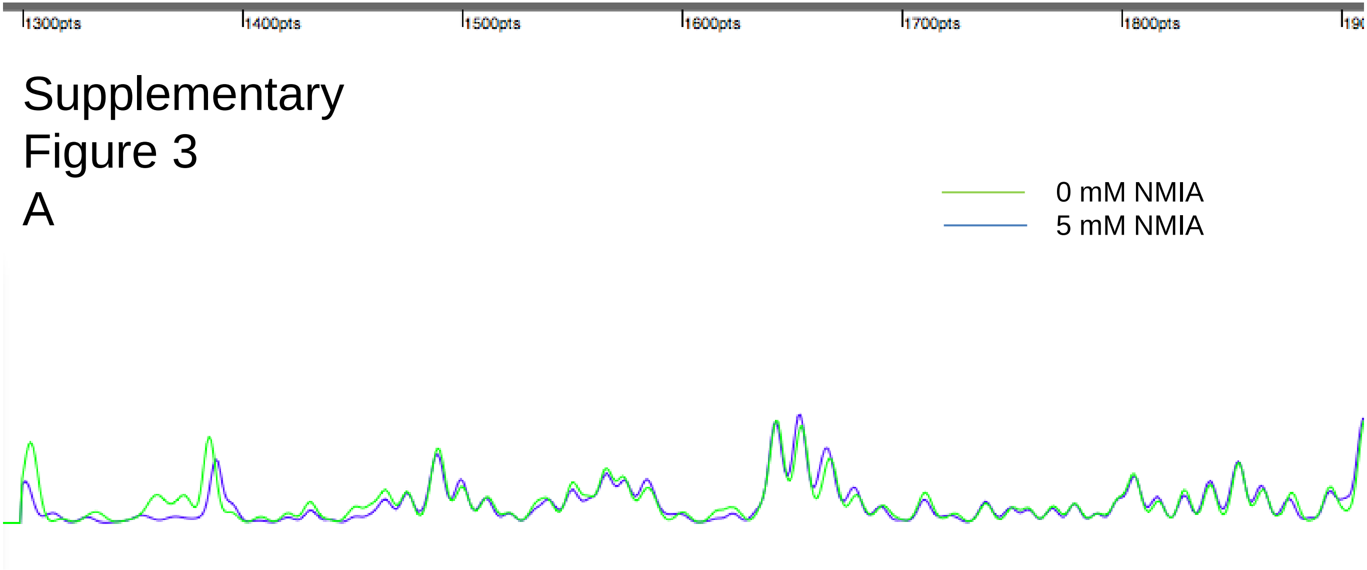

B

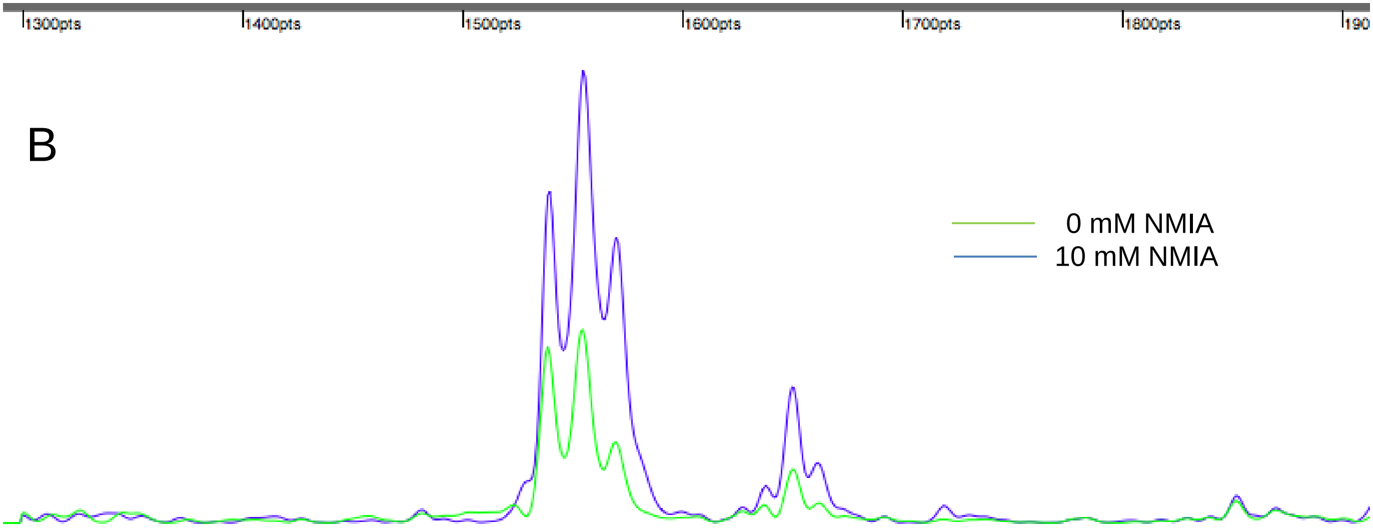

Supplementary Figure 4

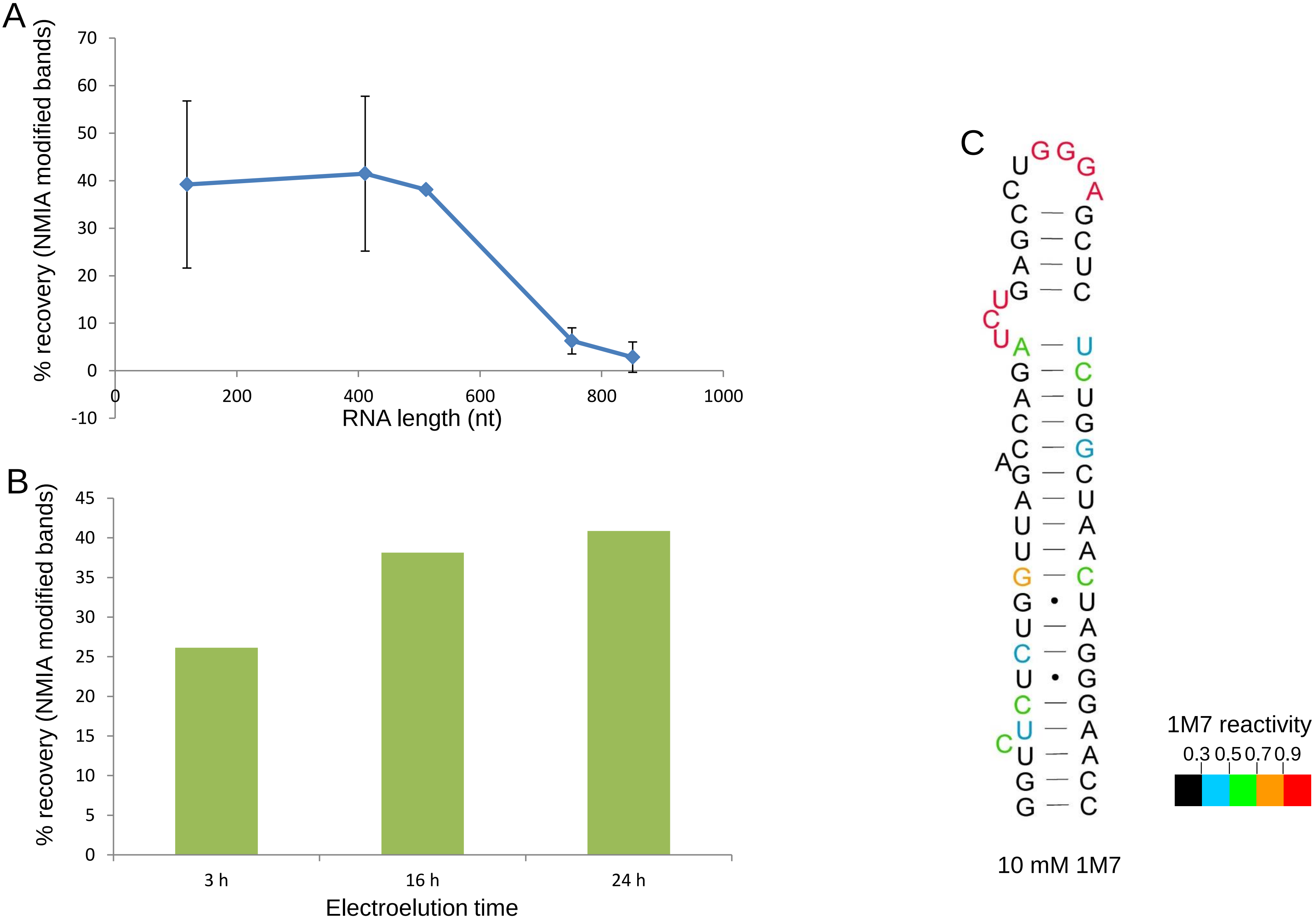

Supplementary Figure 5: NMIA reactivities of monomer and dimer RNA

	Monomer	Dimer
G	0.050012	
G	0.026973	
T	0.022387	
T	0.057819	0.122292
A	0.003316	-0.00053
G	0.032013	0.009658
A	0.410248	0.109366
C	0.011444	0.123648
C	0.696318	0.633783
A	0.275101	-0.02312
G	-0.03475	0.023137
A	0.135949	0.032967
T	0.610516	0.422801
C	2.066889	0.914024
T	0.501995	0.96953
G	0.52876	0.113357
A	0.056429	0.028098
G	-0.00623	0.006441
C	0.025678	0.019638
C	0.046356	0.296877
T	0.586519	0.995093
G	0.880398	1.540878
G	0.344382	1.369417
G	0.003122	1.080919
A	0.575649	0.476933
G	0.132787	0.047973
C	0.006791	-0.01139
T	0.127483	0.068396
C	0.31829	0.134032
T	0.604182	0.287528
C	0.462419	0.203888
T	0.40636	0.229233
G	0.182119	0.140871
G	0.065704	0.021012
C	-0.00183	0.024657
T	0.092777	0.085811
A	0.11261	0.036614
A	0.056941	0.007008
C	0.028243	-0.01697
T	0.280731	0.172235

A	0.122575	-0.00777
G	0.055669	-0.08937
G	0.256371	0.138824
G	1.57651	0.223023
A	1.715056	-0.04804
A	7.500576	0.900708
C	0.221244	0.039322
C	1.659729	0.306379
C	3.547142	0.572446
A	0.065727	-0.03484
C	0.044541	0.140901
T	2.152927	0.747892
G	-0.07774	-0.02467
C	0.023761	-0.00087
T	0.16654	0.024603
T	1.478452	1.268416
A	0.386779	0.070321
A	0.515158	-0.00084
G	0.050201	0.000672
C	0.020586	0.029314
C	0.071671	0.046894
T	0.242402	0.173044
C	0.280921	0.390683
A	0.337105	0.121911
A	0.449676	0.417013
T	1.198138	1.115011
A	0.452213	0.216972
A	0.375539	0.139354
A	0.274332	0.08668
G	0.080522	0.067549
C	0.034882	0.051043
T	0.139172	0.319262
T	0.791708	0.686168
G	0.064628	0.255019
C	0.028088	0.229554
C	0.130644	1.034918
T	0.33012	2.188678
T	1.369887	0.616413
G	0.201716	0.130366
A	0.032028	0.006938
G	0.055391	0.107553
T	0.0487	0.24662
G	0.004542	0.077967

C	0.07004	0.032883
T	0.034176	0.090801
T	0.170994	0.415467
C	0.82183	0.759588
A	0.322611	0.123547
A	0.134036	0.000644
G	0.168378	-0.00759
T	0.037073	0.325397
A	0.111371	-0.00965
G	0.11413	0.038585
T	0.072783	0.501418
G	0.205469	0.727504
T	0.106573	1.193668
G	0.249566	1.00292
T	0.458603	0.539962
G	0.187025	0.233429
C	0.20511	0.29295
C	0.287177	0.110302
C	0.761959	0.263209
G	0.025502	0.005258
T	0.105989	0.262278
C	0.407248	0.577947
T	1.289725	0.613924
G	0.41498	0.189946
T	0.316505	0.121637
T	0.504074	0.563199
G	0.121299	0.199738
T	0.183308	0.486776
G	-0.00171	0.130398
T	0.158051	0.493002
G	0.113274	0.123214
A	0.115249	0.075517
C	0.03118	0.016754
T	0.050153	0.187514
C	0.20904	0.459667
T	0.098271	0.251041
G	0.010016	0.099949
G	-0.05765	-0.00184
T	0.256897	0.977116
A	0.376762	0.318516
A	0.291753	0.089309
C	0.14235	0.060161
T	0.520679	0.417769

A	0.181516	0.087942
G	0.076825	0.068835
A	0.006204	-0.00027
G	0.023414	0.020757
A	0.031467	-0.01035
T	0.562415	0.330777
C	0.982811	0.59104
C	0.402952	0.275028
C	0.143523	0.062955
T	0.219975	0.052908
C	0.530252	0.491058
A	0.018813	-0.01172
G	0.066802	-0.07611
A	3.171926	2.423076
C	4.008308	3.234301
C	6.865119	4.657295
C	10.37569	6.114415
T	2.81494	1.791834
T	0.250413	0.109907
T	2.326168	1.687923
T	2.391118	4.382725
A	0.195177	0.224966
G	-0.01458	0.153532
T	0.12248	-0.15933
C	1.663742	2.579622
A	0.250688	0.057309
G	0.27418	0.10906
T	0.004898	0.126055
G	0.442716	0.352357
T	1.013082	0.66354
G	0.574558	0.75802
G	0.18325	0.239148
A	0.686052	0.284733
A	0.268043	0.047531
A	0.659292	0.317387
A	0.117058	0.074681
T	0.141274	0.120273
C	0.53285	0.523277
T	0.604888	0.637732
C	0.341073	0.288093
T	0.49972	0.501248
A	0.072284	0.035061
G	0.330197	0.452094

C	3.186068	2.173887
A	0.458945	0.125614
G	0.360963	0.036011
T	0.489761	0.164096
G	0.536353	0.154081
G	0.756464	0.128814
C	0.24698	0.027791
G	0.260016	0.075824
C	0.038528	0.057292
C	0.276798	0.315964
C	0.186973	0.54871
G	0.13811	0.407029
A	0.400125	0.106256
A	0.735127	0.60135
C	2.869756	2.245714
A	0.303728	0.09226
G	0.072099	0.03981
G	1.31094	1.506794
G	0.971599	1.083617
A	0.373249	0.061964
C	0.616412	0.564378
C	2.637449	1.751511
T	2.429272	1.883026
G	0.947152	1.068268
A	0.238891	0.11972
A	0.233513	0.148597
A	0.484832	0.340298
G	0.529611	0.697374
C	1.258938	1.280637
G	0.293795	0.26461
A	0.305787	0.133722
A	0.252247	0.110168
A	0.575597	0.273482
G	0.635593	0.465317
G	0.606398	0.613642
G	0.489742	0.442143
A	0.329552	0.123934
A	0.552493	0.151501
A	0.114264	0.061787
C	0.07238	0.141886
C	0.37656	1.561118
A	0.17469	0.291248
G	0.035501	0.259365

A	0.114392	0.167939
G	-0.21821	0.211465
G	0.265266	0.561497
A	-0.00354	0.157471
G	0.16889	0.207515
C	0.010369	0.021056
T	0.569925	0.241624
C	1.426328	0.555028
T	2.383986	1.011575
C	4.701329	0.853016
T	13.8068	1.153867
C	1.90525	0.230092
G	-0.09904	-0.01623
A	-0.03434	0.131281
C	-0.06511	0.098117
G	0.471064	0.046375
C	3.837749	4.274134
A	0.485239	0.353347
G	0.308627	1.93813
G	0.049382	0.951599
A	0.223149	0.22986
C	0.134063	0.005677
T	0.448533	0.008614
C	1.382287	0.269204
G	0.902848	-0.0324
G	0.16098	0.18498
C	0.203685	0.01001
T	0.948423	0.103221
T	1.453144	0.166027
G	-0.00037	-0.00275
C	0.191521	0.090823
T	0.542901	0.311238
G	0.07369	0.196069
A	0.210152	0.502372
A	0.071821	0.493795
G	0.111106	0.109302
C	0.059753	0.013584
G	0.26282	0.021265
C	0.186179	0.004909
G	0.094886	-0.0241
C	0.162903	0.108179
A	0.084277	0.053047
C	0.178129	0.015743

G	-0.021	-0.06638
G	0.022818	-0.03657
C	0.300927	0.151609
A	0.022178	0.062278
A	0.182809	0.370135
G	0.121829	-0.00572
A	0.425452	0.158768
G	0.699063	1.910384
G	0.568286	1.499181
C	1.576917	0.592529
G	0.187401	0.007792
A	0.32464	-0.00864
G	0.011722	-0.05322
G	-0.02264	0.004743
G	0.144115	0.576073
G	0.34779	0.663885
C	0.540704	0.381321
G	0.730114	0.779394
G	1.012827	0.79185
C	1.954317	1.556049
G	0.317782	0.279609
A	0.218891	0.094024
C	0.17623	0.222794
T	0.714102	0.713259
G	0.301111	0.152922
G	0.367974	0.162389
T	0.606275	0.297178
G	0.476713	0.521914
A	0.081681	0.019961
G	0.443435	0.21885
T	0.475469	0.76987
A	0.162796	0.132292
C	0.209182	0.026184
G	0.985166	0.829504
C	1.127916	0.696832
C	1.136573	0.73826
A	0.323252	0.219462
A	0.207345	0.115746
A	0.284386	0.21613
A	0.559923	0.3995
A	0.729957	0.593495
T	0.396221	0.577844
T	1.110475	1.097376

T	0.95898	0.513304
T	1.641975	1.621979
G	0.09999	0.552409
A	0.188692	0.097016
C	0.117734	0.065947
T	0.194971	0.434695
A	0.149432	0.000123
G	0.029363	-0.00306
C	0.114023	0.039744
G	0.324384	0.002364
G	0.675526	1.372586
A	0.792314	0.665699
G	0.874052	0.890554
G	0.145622	0.09315
C	0.257896	0.221177
T	0.101083	0.150211
A	0.101698	0.112634
G	0.127334	0.040598
A	0.175311	0.137196
A	0.196934	0.127976
G	0.662018	0.578286
G	0.425736	0.41222
A	0.206993	0.138112
G	0.114217	0.139967
A	0.635699	0.293651
G	0.423786	0.561158
A	0.435373	0.238471
G	0.343667	0.279714
A	0.245296	0.193836
U	0.48852	0.59131
G	0.280689	-0.06128
G	0.106717	-0.03898
G	0.447891	0.301656
U	0.331455	0.466703
G	-0.00105	0.039489
C	0.200343	0.256638
G	0.207243	0.161087
A	0.275339	0.045416
G	0.556786	0.482759
A	0.797652	0.645631
G	0.152898	0.113385
C	0.266031	0.246848
G	0.288627	-0.00886

U	0.451224	2.851655
C	0.959521	15.6831
A	0.140483	0.239215
G	1.786214	2.730999
U	-0.21032	19.97955
A	0.429156	

Supplementary Figure 6

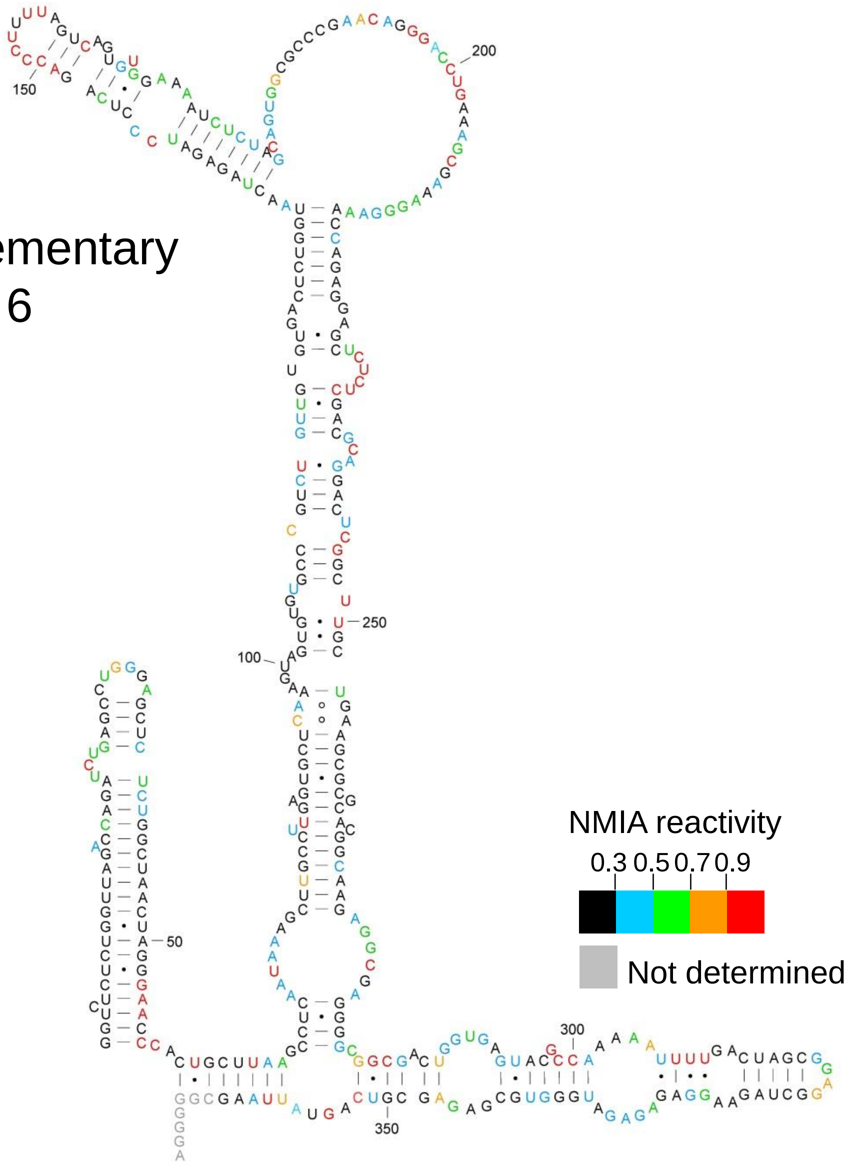

Supplementary Figure legends

Supplementary Figure 1: Schematic representation of sample preparation for in-gel probing. See Materials and Methods for experimental details.

Supplementary Figure 2: Numerical NMIA reactivity data for TAR RNA.

Supplementary Figure 3: Optimisation of NMIA concentration. SHAPEfinder reactivity traces of TAR RNA modified in-gel with DMSO only (green) or with 5 mM or 10 mM NMIA (A and B respectively). The overlapping nature of the blue and green traces in A shows that RNA is not modified significantly above control levels using 5 mM NMIA. In contrast, 10 mM NMIA shows reactivity at two main locations (B).

Supplementary Figure 4: Optimisation of RNA recovery and use of further SHAPE reagents. A, graph showing percentage recovery of RNA from the NMIA modified bands. Retroviral RNAs of 118, 411, 511, 751 and 851 nt were transcribed, renatured, electrophoresed, in-gel probed with 10 mM NMIA and electroeluted as described in Materials and Methods, and the percentage recovery determined by spectrophotometry. Data points are an average of 3-4 replicates, except for 511nt, which was a single replicate. B, bar graph illustrating the percentage recovery of RNA as a function of electroelution time. A 511 nt RNA was transcribed, renatured, fractionated, in-gel probed with 10 mM NMIA as described in Materials and Methods and electroeluted for the indicated times. Recovery was determined by spectrophotometry. C, 1M7 reactivities of nucleotides in TAR, in-gel probed with 10 mM 1M7 for 3 min at 37°C. Reactivity values are colour-coded as indicated by the key.

Supplementary Figure 5: Numerical NMIA reactivity data for HIV-1 monomer and dimer.

Supplementary Figure 6: In-gel SHAPE data for monomeric RNA mapped onto the LDI structural model. Data are from the experiments illustrated in Figure 5 and Supplementary Figure 5. Reactivities are colour-coded as shown in the key.
